# Curcumin Ameliorates Methotrexate-Induced Nephrotoxicity in Rats

**DOI:** 10.1155/2013/387071

**Published:** 2013-12-05

**Authors:** Mohamed A. Morsy, Salwa A. Ibrahim, Entesar F. Amin, Maha Y. Kamel, Rehab A. Rifaai, Magdy K. Hassan

**Affiliations:** ^1^Department of Pharmacology, Faculty of Medicine, Minia University, 61511 El-Minia, Egypt; ^2^Department of Histology, Faculty of Medicine, Minia University, 61511 El-Minia, Egypt; ^3^Department of Physiology, Faculty of Medicine, Minia University, 61511 El-Minia, Egypt

## Abstract

Methotrexate is an effective anticancer and immunosuppressive agent. However, nephrotoxicity is one of the complications of its use. On the other hand, curcumin, a naturally occurring polyphenolic compound, is reported to have antioxidant and anti-inflammatory properties. Those two properties are likely to prevent methotrexate-induced nephrotoxicity. The aim of this study is to evaluate the possible protective effect of curcumin against methotrexate-induced nephrotoxicity and delineate various mechanism(s) underlies this effect in rats. Nephrotoxicity was induced in Wistar rats by intraperitoneal administration of methotrexate (7 mg/kg/day) for three consecutive days. Curcumin administration in methotrexate-intoxicated rats resulted in nephroprotective effects as evidenced by the significant decrease in levels of serum creatinine and urea as well as renal malondialdehyde, nitric oxide, and tumor necrosis factor-**α** with a concurrent increase in renal glutathione peroxidase and superoxide dismutase activities compared to nephrotoxic untreated rats. Additionally, immunohistochemical analysis demonstrated that curcumin treatment markedly reduced cyclooxygenase-2 expression. Histopathological examination confirmed the protective effects of curcumin. In conclusion, curcumin protected rats from methotrexate nephrotoxicity, at least in part, through its antioxidant and anti-inflammatory activities.

## 1. Introduction

Methotrexate, a folic acid antagonist, is widely used in the treatment of various malignancies and inflammatory diseases. However, nephrotoxicity is an important adverse effect of methotrexate therapy [1]. The pathogenesis of methotrexate nephrotoxicity involves multiple pathways, including oxidative stress and inflammation [2, 3]. Several agents have been used, with various degrees of success, to ameliorate or prevent methotrexate nephrotoxicity [2–4].

Curcumin is an active polyphenolic constituent from *Curcuma longa *with notable antioxidant and anti-inflammatory properties [5, 6] that render it an attractive candidate for protection against methotrexate nephrotoxicity. Curcumin has shown renal protective properties against gentamicin- and cisplatin- induced renal toxicities ([7] and [8], resp.) as well as diabetic nephropathy [9]. The present study therefore was designed to assess the possible renoprotective effect of curcumin and to examine the underlying mechanism(s) responsible for this effect in a rat model of methotrexate-induced nephrotoxicity. The mechanism of renoprotection was evaluated by assessing the oxidative stress (i.e., malondialdehyde, nitric oxide, glutathione peroxidase, and superoxide dismutase) and inflammatory (i.e., tumor necrosis factor-*α* [TNF-*α*] and cyclooxygenase-2 [COX-2]) parameters.

## 2. Materials and Methods

### 2.1. Chemicals

Curcumin was a generous gift from DBK Pharma (Cairo, Egypt). Methotrexate was a generous gift from Minapharm (Cairo, Egypt). Antibody against COX-2 was purchased from Thermo Fisher Scientific Inc./Lab Vision (Fremont, CA, USA). All other chemicals were of analytical grade and were obtained from commercial sources.

### 2.2. Animals and Experimental Design

Ten-week-old male Wistar rats weighing 150–180 g were used after one week for proper acclimatization to the animal house conditions (12 h lighting cycle and 25 ± 2°C temperature) with free access to standard rodent chow and water. All experimental procedures were conducted according to the ethical standards approved by the Institutional Animal Ethics Committee guidelines for animal care and use, Minia University, Egypt. Animals were randomly divided into four groups (6–8 animals each). The first group served as the control group. The second group was treated with curcumin (200 mg/kg p.o.) [10] suspended in 1% aqueous solution of carboxymethyl cellulose daily for three consecutive days and served as positive control. The third group was injected with methotrexate (7 mg/kg, i.p.) [11] daily for three consecutive days to induce nephrotoxicity. The fourth group was given curcumin (200 mg/kg, p.o.) 1 h before methotrexate (7 mg/kg, i.p.) daily for three consecutive days. All groups received equivalent volumes of the used vehicles. Rats were sacrificed 24 h after the last methotrexate injection, and blood samples were collected and centrifuged at 3000 ×g for 10 min to obtain clear sera. The longitudinal section of the left kidney was excised from each animal for histological and immunohistochemical examination. The renal cortex of the rest of the kidneys were snap frozen in liquid nitrogen, stored at −80°C, and subsequently homogenized in cold potassium phosphate buffer (0.05 M, pH 7.4) for various biochemical analyses.

### 2.3. Biochemical Analysis

Using commercially available kits, serum levels of creatinine and urea (Diamond Diagnostics, Egypt) as well as renal glutathione peroxidase and superoxide dismutase (Biodiagnostic, Egypt) activities were quantified according to the manufacturers' guidelines. Renal TNF-*α* assay was performed with rat TNF-*α* ELISA kit (RayBiotech, Inc., GA, USA) according to supplier's instructions. Renal cortex lipid peroxidation was determined as thiobarbituric acid reacting substance and is expressed as equivalents of malondialdehyde, using 1,1,3,3-tetramethoxypropane as standard [12]. Renal cortex nitric oxide level was measured as total nitrite/nitrate, the stable degradation products of nitric oxide, by reduction of nitrate into nitrite using copperized cadmium, followed by color development with Griess reagent in acidic medium [13].

### 2.4. Histological and Immunohistochemical Examination

Renal tissue samples were fixed in 10% neutral buffered formalin, embedded in paraffin, sectioned, and stained with hematoxylin and eosin for histological examination using light microscopy. Three sections from each animal group were subjected to semiquantitative microscopical analysis using light microscopy (Olympus CX41). Renal changes were graded as mild, moderate, or severe. Scores +, ++, and +++ are mild, moderate, and severe levels, revealing less than 25, 50, and 75% histopathological alterations of total fields examined, respectively.

For immunohistochemical detection of COX-2 expression, UltraVision ONE HRP polymer detection system (Thermo Fisher Scientific Inc./Lab Vision, Fremont, CA, USA) was used according to the manufacturer's protocol. Briefly, kidney sections were deparaffinized and rehydrated. Nonspecific binding of IgG was blocked using Ultra V block for 10 min at room temperature. The sections were then incubated with ready-to-use monoclonal COX-2 antibody for 1 h. After three washes, the sections were incubated for further 30 min with UltraVision One HRP polymer. Color reaction was developed by incubation with diaminobenzidine. The slides were then counterstained, dehydrated, and mounted.

### 2.5. Statistical Analysis

The data are expressed as means ± SEM. Statistical analysis was performed by one-way ANOVA followed by Tukey-Kramer postanalysis test for multiple comparisons with *P* < 0.05 being considered as statistically significant.

## 3. Results

### 3.1. Effects of Curcumin on Renal Functions

**Figure 1:**
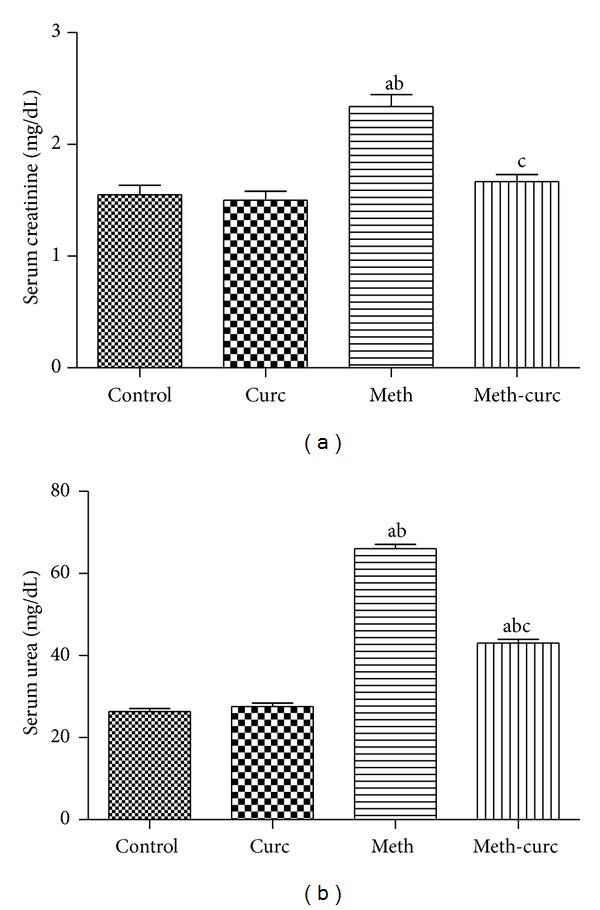
Effect of curcumin (Curc) on serum creatinine (a) and urea (b) levels of methotrexate- (Meth-) induced nephrotoxicity in rats. Data are mean ± SEM of 6–8 rats. ^a,b,c^Significantly different from control, Curc, and Meth groups, respectively, at *P* < 0.05.

Serum creatinine and urea levels were assessed as markers of renal functions. Curcumin treatment in methotrexate-intoxicated rats significantly decreased serum creatinine and urea levels compared to methotrexate alone treated rats. On the other hand, curcumin alone did not alter renal function markers (Figures 1(a) and 1(b)).

### 3.2. Effects of Curcumin on Renal Histopathological Changes

**Figure 2:**
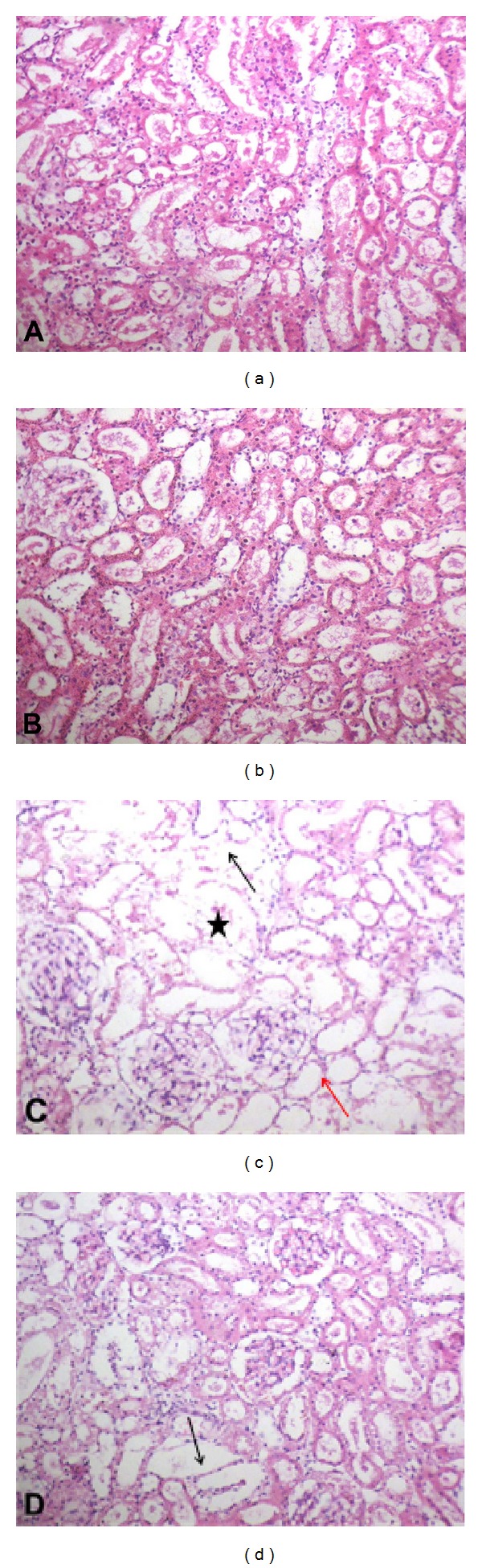
Effect of curcumin on kidney histopathological picture of methotrexate-induced nephrotoxicity in rats (H&E ×200). ((a) and (b)) Sections from control and curcumin-treated groups show normal histological pattern. (c) Methotrexate-treated group shows degeneration of the renal tubules with disruption of the basement membranes in-between the tubules (black arrow). Most of the renal tubules show cystic luminal dilatation and their lining cells are flat (red arrow). Degenerated glomeruli are also observed (star). (d) Kidney tissue from methotrexate plus curcumin treated rats; the histological features in section (c) are greatly improved and nearly back to normal appearance. Still few tubules were dilated (arrow).

Histopathological changes were screened to support the results of the classical markers of renal functions. Both control and curcumin-treated groups showed normal histological pattern. Histopathological examination revealed that injection of methotrexate produced degeneration of renal tubules that showed cystic luminal dilatation as compared with control group. Curcumin administration was able to restore methotrexate-induced histopathological damage (Figures 2(a)–2(d); Table 1).

### 3.3. Effects of Curcumin on Renal Malondialdehyde, Nitric Oxide, Glutathione Peroxidase, and Superoxide Dismutase

**Figure 3:**
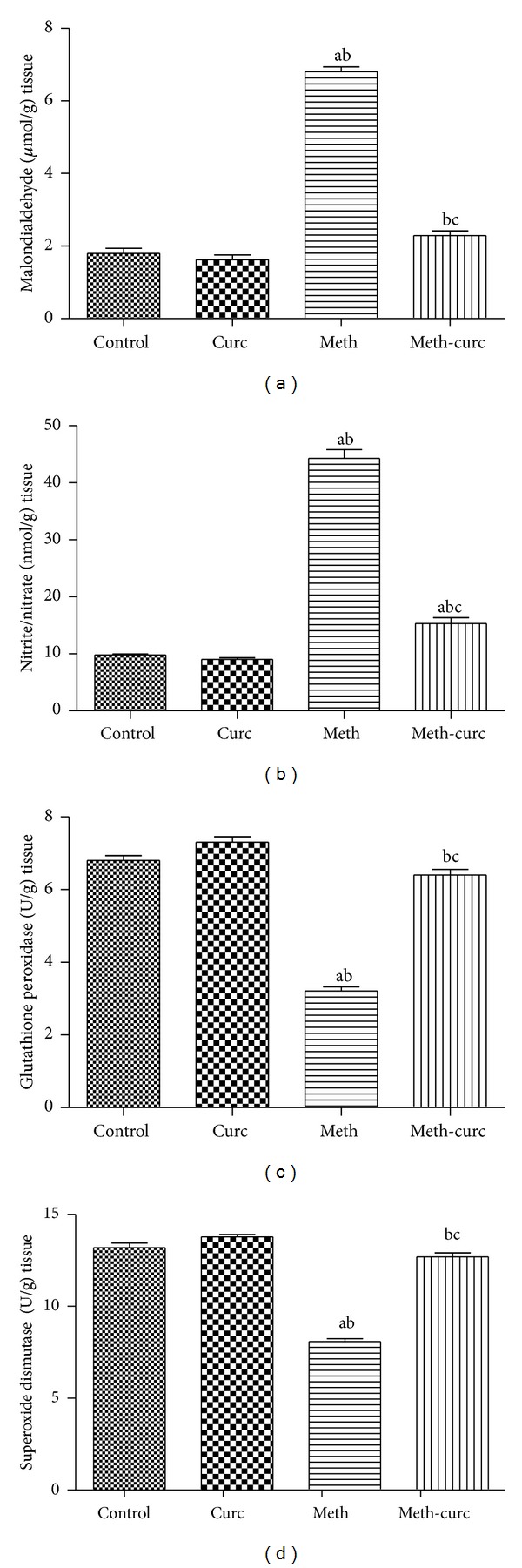
Effect of curcumin (Curc) on renal malondialdehyde (a), nitric oxide (as nitrite/nitrate; (b)), glutathione peroxidase (c), and superoxide dismutase (d) levels of methotrexate- (Meth-) induced nephrotoxicity in rats. Data are mean ± SEM of 6–8 rats. ^a,b,c^Significantly different from control, Curc, and Meth groups, respectively, at *P* < 0.05.

Oxidative stress was assessed through measuring renal malondialdehyde and nitrite/nitrate levels as well as glutathione peroxidase and superoxide dismutase activities. Renal malondialdehyde was evaluated as an indicator of renal lipid peroxidation and nitrite/nitrate as an indicator of renal nitric oxide level. Curcumin treatment significantly suppressed both lipid peroxidation and the elevation of NO levels in comparison with methotrexate-intoxicated group (Figures 3(a) and 3(b)). Moreover, curcumin treatment significantly increased renal glutathione peroxidase and superoxide dismutase activities compared to methotrexate-treated group (Figures 3(c) and 3(d)).

### 3.4. Effects of Curcumin on TNF-*α* Level and COX-2 Expression

**Figure 4:**
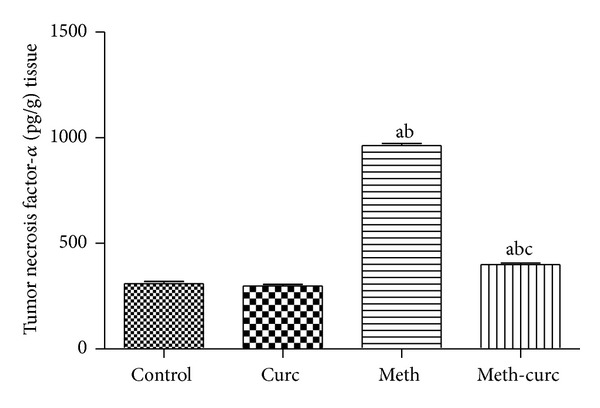
Effect of curcumin (Curc) on renal tumor necrosis factor-*α* level of methotrexate- (Meth-) induced nephrotoxicity in rats. Data are mean ± SEM of 6–8 rats. ^a,b,c^Significantly different from control, Curc, and Meth groups, respectively, at *P* < 0.05.

**Figure 5:**
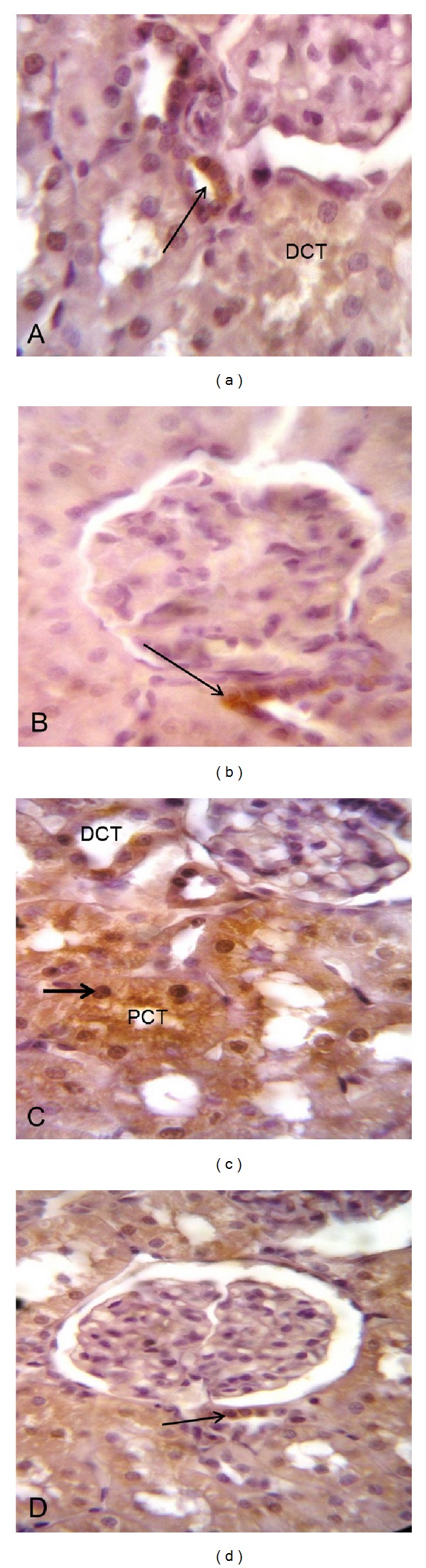
Effect of curcumin on localization of cyclooxygenase-2 (COX-2) immunoreactivity in rat renal cortex of methotrexate-induced nephrotoxicity in rats (×1000). ((a) and (b)) Sections from control and curcumin-treated groups show localized COX-2 expression in the macula densa cells (arrow). (c) Methotrexate-treated group shows high COX-2 expression in different degenerated cortical tubules. Note that some nuclei show positive COX-2 staining (arrow). (d) Methotrexate plus curcumin treated group shows little COX-2 staining in different cortical tubules. Note that COX-2 staining in the macula densa cells (arrow). PCT: proximal convoluted tubule; DCT: distal convoluted tubule.

**Figure 6:**
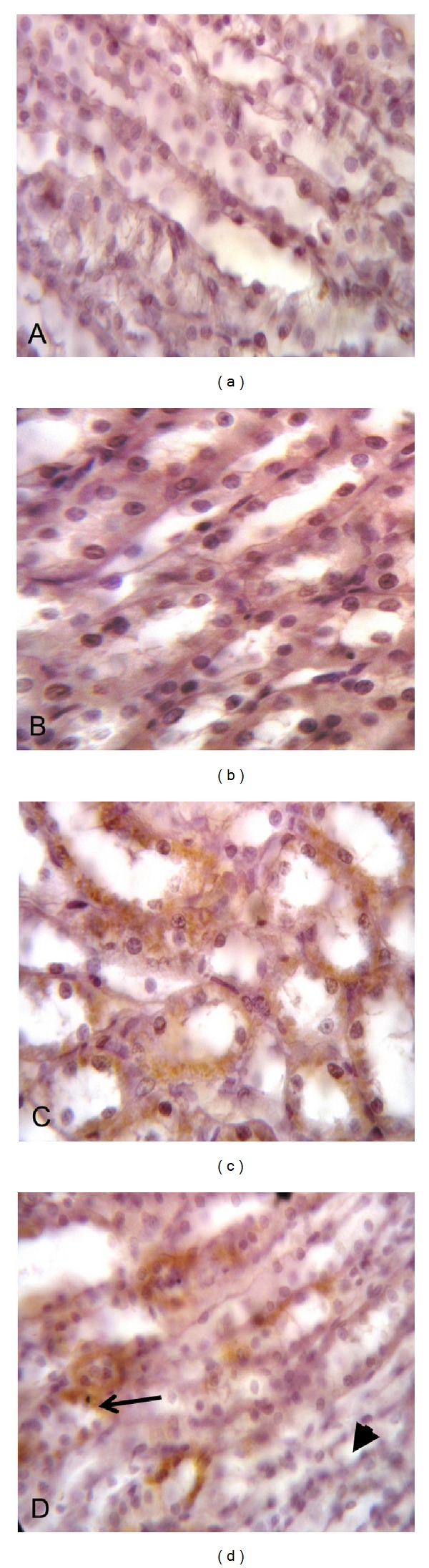
Effect of curcumin on localization of cyclooxygenase-2 (COX-2) immunoreactivity in rat renal medulla of methotrexate-induced nephrotoxicity in rats (×1000). ((a) and (b)) Sections from control and curcumin-treated groups show absence of COX-2 expression in the medullary tubules. (c) Methotrexate-treated group shows most of the medullary tubules exhibiting COX-2 staining. (d) Methotrexate plus curcumin treated group shows some medullary tubules displaying COX-2 expression (arrow) while others show absence of COX-2 expression (arrowhead).

The inflammatory mediators TNF-*α* and COX-2 were assessed. Curcumin treatment significantly decreased the elevation of TNF-*α* levels in comparison with methotrexate-intoxicated group (Figures 4(a)–4(d)). Immunohistochemical staining of rat kidney showed that COX-2 protein was expressed in renal cortex mainly in the macula densa cells of some distal tubule in the control and curcumin treated groups (Figures 5(a) and 5(b)). Negative expression was noticed in the renal medulla (Figures 6(a) and 6(b)). In methotrexate-treated group, high COX-2 expression was observed in different degenerated cortical tubules (proximal and distal convoluted tubules). Interestingly, in some tubular cells, there was translocation of the expression from the cytoplasm to the nucleus (Figure 5(c)). Most of the medullary tubules exhibited COX-2 staining (Figure 6(c)). COX-2 staining was diminished in the methotrexate and curcumin treated group both in the renal cortex (Figure 5(d)) and renal medulla (Figure 6(d)).

## 4. Discussion

Curcumin exerts a variety of pleiotropic effects including antioxidant and anti-inflammatory actions, which should facilitate better protection from methotrexate nephrotoxicity. In the present study, curcumin significantly decreased serum creatinine as well as urea levels and attenuated histopathological alterations in methotrexate-intoxicated rats. Consistent with these results, treatment with curcumin significantly decreased creatinine as well as blood urea nitrogen levels and reduced histopathological changes associated with 5/6 nephrectomized rats [14] and cisplatin-induced nephrotoxicity [8].

Since oxidative stress plays an important role in the development of methotrexate nephrotoxicity, several oxidative stress parameters were therefore assessed. In present study, the ability of curcumin to increase renal glutathione peroxidase and superoxide dismutase activities is in line with the finding of Tapia et al. [14] who found that curcumin prevented the decrease in the activity of antioxidant enzymes including glutathione peroxidase and superoxide dismutase in rat remnant kidney. On the other hand, in agreement with the current study, several previous studies [5, 14] denoted similar findings concerning the ability of curcumin to decrease lipid peroxidation in rats subjected to 5/6 nephrectomy. This inhibitory effect of curcumin on lipid peroxidation could be secondary to its antioxidant activity. Alternatively, in harmony with our study, curcumin decreased renal nitric oxide levels in gentamicin- and cholestasis- induced renal injury ([7] and [15], resp.). Christo et al. [16] reported that nitric oxide has a role in the acute renal failure because of the fact that the free radical nature of nitric oxide might contribute to tubular damage. Additionally, nitric oxide increases renal injury through its reaction with superoxide radical and generation of a cytotoxic peroxynitrite [17], which could damage the tubular cells resulting in renal failure. The decrease in nitric oxide level may be due to decrease in inducible nitric oxide synthase level as curcumin is reported to reduce it [7]. Moreover, sustained inducible nitric oxide synthase-mediated nitric oxide generation may mediate lipid peroxidation [18]. In addition, oxidative stress is known to stimulate transcription factors, including nuclear factor-*κ*B (NF-*κ*B) [19]. Meanwhile, NF-*κ*B is known to activate many genes, including iNOS [20].

Inflammatory mediators including TNF-*α* and COX-2 play important roles in the pathogenesis of methotrexate nephrotoxicity. In accordance with the present study, the reduced renal TNF-*α* level and COX-2 expression in curcumin-treated methotrexate group is strengthened by several studies in rat kidney [5, 21]. TNF-*α* stimulates the production of other inflammatory mediators including COX-2 along with inducible nitric oxide synthase and curcumin can reduce the levels of these mediators [6].

## 5. Conclusions

Curcumin treatment attenuated methotrexate nephrotoxicity in rats partly through its antioxidant and anti-inflammatory properties by preserving glutathione peroxidase and superoxide dismutase activities and inhibiting TNF-*α* and COX-2 production.

## Figures and Tables

**Table 1:** Effect of curcumin on the severity of histopathological lesions in methotrexate- (MTX-) induced nephrotoxicity in rats.

Groups	Tubular degeneration	Tubular dilatation	Glomerular degeneration
Control	0	0	0
Curcumin	0	0	0
MTX	+++	+++	+
MTX + curcumin	+	+	0

Score level 0 was considered normal. Scores +, ++, and +++ are mild, moderate, and severe levels, revealing less than 25, 50 and 75% histopathological alterations of total fields examined, respectively. Score represents values obtained from tissue sections of 3 animals of each group.
